# Immune Condition of Colorectal Cancer Patients Featured by Serum Chemokines and Gene Expressions of CD4+ Cells in Blood

**DOI:** 10.1155/2018/7436205

**Published:** 2018-06-11

**Authors:** Takuya Komura, Masaaki Yano, Akimitsu Miyake, Hisashi Takabatake, Masaki Miyazawa, Norihiko Ogawa, Akihiro Seki, Masao Honda, Takashi Wada, Shigeyuki Matsui, Shuichi Kaneko, Yoshio Sakai

**Affiliations:** ^1^System Biology, Graduate School of Advanced Preventive Medical Sciences, Kanazawa University, Japan; ^2^Disease Control and Homeostasis, College of Medical Pharmaceutical and Health Sciences, Kanazawa University, Japan; ^3^Department of Biostatistics, Nagoya University Graduate School of Medicine, Japan; ^4^Department of Gastroenterology, Kanazawa University Hospital, Kanazawa, Japan; ^5^Department of Nephrology, Kanazawa University Hospital, Kanazawa, Japan

## Abstract

**Background:**

Colorectal cancer (CRC), the most common malignancy worldwide, causes inflammation. We explored the inflammatory pathophysiology of CRC by assessing the peripheral blood parameters.

**Methods:**

The differences in gene expression profiles of whole blood cells and cell subpopulations between CRC patients and healthy controls were analyzed using DNA microarray. Serum cytokine/chemokine concentrations in CRC patients and healthy controls were measured via multiplex detection immunoassays. In addition, we explored correlations between the expression levels of certain genes of peripheral CD4+ cells and serum chemokine concentrations.

**Results:**

The gene expression profiles of peripheral CD4+ cells of CRC patients differed from those of healthy controls, but this was not true of CD8+ cells, CD14+ cells, CD15+ cells, or CD19+ cells. Serum IL-8 and eotaxin-1 levels were significantly elevated in CRC patients, and the levels substantially correlated with the expression levels of certain genes of CD4+ cells. Interestingly, the relationships between gene expression levels in peripheral CD4+ cells and serum IL-8 and eotaxin-1 levels resembled those of monocytes/macrophages, not T cells.

**Conclusions:**

Serum IL-8 and eotaxin-1 concentrations increased and were associated with changes in the gene expression of peripheral CD4+ cells in CRC patients.

## 1. Introduction

Colorectal cancer (CRC) is the third most common fatal malignancy worldwide [1]. It is important to detect CRC early to improve prognosis [2, 3]. Currently, the fecal occult blood test (FOBT) for in vitro diagnostic use is used to screen for CRC; however, the positive predictive rate is poor [3]. Colonoscopy is superior, but this is invasive and associated with multiple complications including perforation, pain, and discomfort [4]. Thus, alternative noninvasive diagnostic tests are required. To this end, it is necessary to understand the pathological features, including the immune status, of CRC patients. 

We previously reported that the gene expression profiles of peripheral blood cells from patients with cancers of the digestive system differed from those of noncancerous controls [5]. Peripheral blood contains many types of immune cells including neutrophils, monocytes, and macrophages [6]. Changes in gene expression are hypothesized to reflect the reactions of the immune system to cancer, because cancer is frequently associated with the appearance of various types of inflammatory cells [7]. These include helper T cells and cytotoxic T lymphocytes [8], which inhibit cancer progression, and myeloid-derived suppressor cells [9], regulatory T cells [10], and programmed cell death 1 (PD-1) expressing T cells [11], which promote cancer development. We previously reported that immune response-eliciting or immunosuppressive molecules mediated interactions between circulating peripheral blood cells and local cancer tissues in patients with pancreatic ductal adenocarcinomas [12] and hepatocellular carcinomas [13, 14]. In contrast, the features of immune pathophysiology reflected in peripheral blood of colorectal cancer have yet to be investigated.

Here, we observed that the gene expression profiles of peripheral CD4+ cells and whole blood cells of CRC patients differed from those of healthy controls. The serum concentrations of IL-8 and eotaxin-1 were elevated in CRC patients compared to healthy controls.

## 2. Methods

### 2.1. CRC Patients and Healthy Controls

Blood was drawn from CRC patients prior to treatment and from healthy controls. A total of 30 CRC patients and 28 healthy controls (Supplemental Table 1) provided serum samples for cytokine and chemokine analyses. CRC was clinically staged using the tumor, node, and metastasis staging system of the Union of International Cancer Control (8th edition). Five CRC patients and seven healthy volunteers donated peripheral blood for gene expression analyses (Supplemental Table 2). Serum cytokine/chemokine levels were measured in four CRC patients and five healthy volunteers (Supplemental Table 2). Written informed consent was obtained from all participants. This study was approved by our Institutional Review Board and was performed in accordance with all relevant tenets of the Declaration of Helsinki.

### 2.2. Serum Cytokine and Chemokine Analyses

Serum cytokine and chemokine levels were measured using a Bio-Plex human cytokine 27-plex panel (Bio-Rad, Tokyo, Japan) according to the manufacturer's instructions. This kit was used to detect interleukin- (IL-) 1*β*, IL-ra, IL-2, IL-4, IL-5, IL-6, IL-7, IL-8, IL-9, IL-10, IL-12 (p70), IL-13, IL-15, IL-17, fibroblast growth factor-basic, eotaxin-1, G-CSF, granulocyte-colony stimulating factor, IFN-*γ*, IP-10, monocyte chemotactic protein-1 (MCP-1), macrophage inflammatory protein- (MIP-) 1*α*, MIP-1*β*, platelet-derived growth factor-BB, tumor necrosis factor- (TNF-) *α*, and vascular endothelial growth factor.

### 2.3. Isolation of Peripheral Blood Mononuclear Cells

Peripheral blood mononuclear cells were isolated from heparinized venous blood via Ficoll-Hypaque (Sigma-Aldrich, St. Louis, MO, USA) density gradient centrifugation, as previously described [13]. Then the cells were incubated with bead-labeled anti-CD4, anti-CD8, anti-CD14, or anti-CD15 antibodies (Miltenyi, Cologne, Germany) and isolated using a magnet.

### 2.4. DNA Microarray and Data Analysis

PAXgene® Blood RNA Tubes (PreAnalytiX GmbH, Germany) were used to collect samples for mRNA extraction. Total RNA was isolated from subfractionated peripheral blood cells using a microRNA isolation kit (Stratagene, La Jolla, CA, USA). Isolated RNA was labeled with Cy3 using the Quick-Amp Labeling Kit (Agilent Technologies, Palo Alto, CA, USA) and hybridized to the Whole Human Genome Microarray kit, 4x44K (Agilent Technologies). The slides were scanned using a microarray scanner (Model G2505B; Agilent Technologies), and gene expression analyses were performed using the BRB array tools (NCI, http://linus.nci.nih.gov/BRB-ArrayTools.html). Hierarchical clustering of gene expression data was used to identify differentially expressed genes. Biological processes and networks were analyzed with the aid of the MetaCore® software suite (GeneGo, Carlsbad, CA, USA).

### 2.5. Statistical Analysis

The unpaired Student's* t-*test was used to assess differences between groups, and a p < 0.05 was considered statistically significant. Pearson correlations between IL-8 and eotaxin-1 levels and clinical parameters were calculated. Spearman correlations were derived to explore associations between changes in chemokine concentrations and genes that were differentially expressed in peripheral CD4+ cells of CRC patients and healthy volunteers.

## 3. Results

### 3.1. Serum IL-8 and Eotaxin-1 Levels in CRC Patients

**Figure 1:**
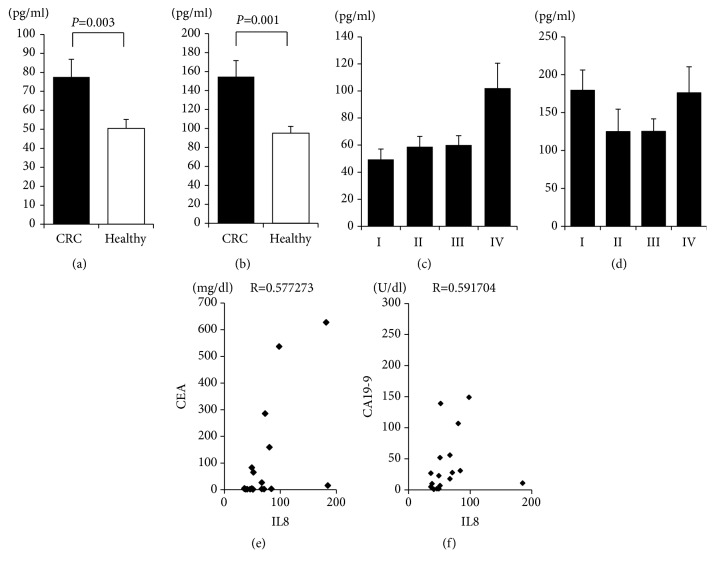
*Serum concentrations of eotaxin-1 and IL-8. *Sera were obtained from CRC patients (n=30) prior to treatment and healthy volunteers (n=28). The serum concentrations of cytokines and chemokines were measured using a multiplex bead immunoassay system. (a) IL-8 levels; (b) eotaxin-1 levels. IL-8 levels in CRC patients by clinical stage (c) and eotaxin-1 levels by clinical stage (d). Correlations between IL-8 and CEA levels in CRC patients (e) and between IL-8 and CA19-9 levels (f).

First, we measured cytokine and chemokine levels in 30 CRC patients and 28 healthy controls using a bead-based multiplex immunoassay (Supplemental Table 1). The serum concentrations of IL-8 and eotaxin-1 were significantly elevated in CRC patients (n=30) compared to healthy controls (n=28) (Figures 1(a) and 1(b)). However, we did not observe an increase of the other proinflammatory cytokines such as TNF-*α*, IFN-*γ*, and IL-12, of a decrease of anti-inflammatory cytokines such as IL-10 (Supplemental Fig.  2).

IL-8 concentrations were only elevated in patients of advanced clinical stage (Stage IV; Figure 1(c)); eotaxin-1 levels did not differ by clinical stage (Figure 1(d)). IL-8 concentrations correlated with those of CEA (Figure 1(e)) and CA19-9 (Figure 1(f)) (r=0.577273 and r=0.591704, respectively), whereas eotaxin-1 concentrations did not (r=0.008045 and r=-0.06421, respectively) (data not shown). No correlation between the level of any other serum cytokine/chemokine and stage or level of tumor marker CEA or CA19-9 was apparent (data not shown).

### 3.2. Gene Expression Profiling of Peripheral Blood Cells from CRC Patients

**Figure 2:**
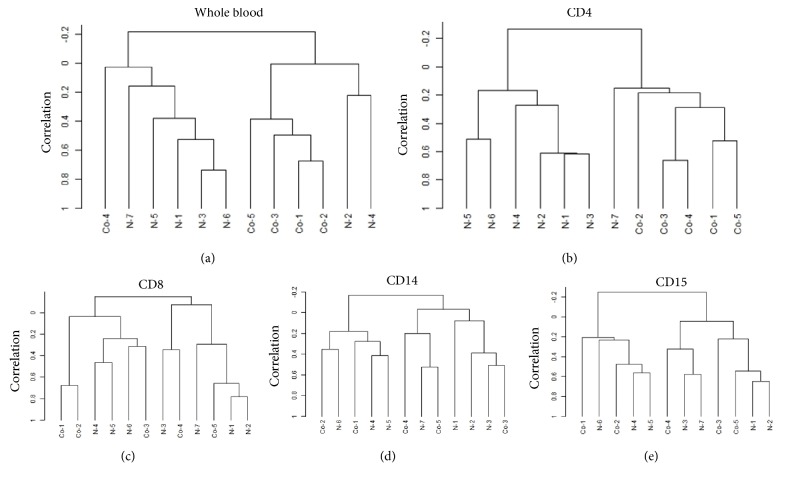
*Unsupervised clustering analysis of the gene expression profiles of subfractionated peripheral blood cells.* The dendrogram used for clustering using employed both correlations and average linkage for unsupervised analysis of the gene expression profile of peripheral blood. Significant up- or downregulated changes in gene expression (≥1.5-fold) in whole blood and CD4+ cells of CRC patients (compared to healthy volunteers) were observed for 3,243 and 2,459 genes at* P*-values <0.05, respectively, but few such changes were observed in CD8+ cells, CD14+ cells, or CD15+ cells (1,475, 128, and 333 genes;* P* <0.05, respectively).

Next, we used DNA microarray to determine if gene expression was altered in peripheral blood cells of five CRC patients and seven healthy volunteers (Supplemental Table 2). Unsupervised clustering analyses revealed a difference in the gene expression patterns of whole blood (Figure 2(a)) and CD4+ cells (Figure 2(b)), but not of CD8+ (Figure 2(c)), CD14+ (Figure 2(d)), or CD15+ cells (Figure 2(e)).

### 3.3. Elevated Serum IL-8 and Eotaxin-1 Concentrations Were Significantly Correlated with Genes Expression, the Levels of Which Were Altered in the Peripheral CD4+ Cells of CRC Patients

**Figure 3:**
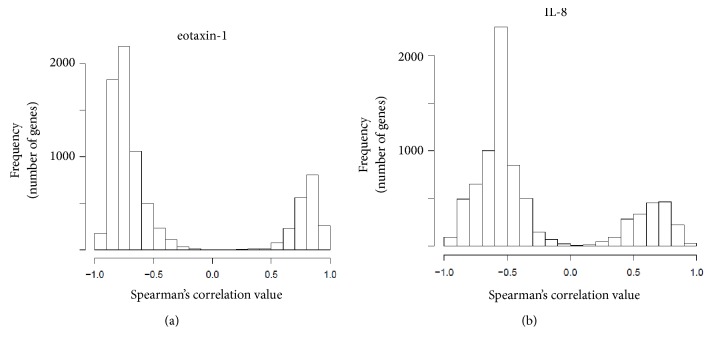
*Spearman correlation coefficients between genes expressed by CD4+ cells. *Genes expressed by CD4+ cells of CRC patients and healthy volunteers, with two-sided p-values, (a) eotaxin-1 and (b) IL-8 levels.

The data described above suggested that expression of the humoral chemokines, eotaxin-1 and IL-8, played a role in the inflammation of CRC patients. In addition, both peripheral CD4+ cells and whole blood cells were affected. Therefore, we derived Spearman's correlations between the serum concentrations of IL-8 and eotaxin-1 and the expression levels of 8,061 genes, the levels of which were altered (FDR<0.05) in CD4+ cells of four CRC patients compared to five healthy volunteers (Supplemental Table 2). We also confirmed that the serum concentrations of IL-8 (Supplemental Fig.  1A), eotaxin-1 (Supplemental Fig.  1B), MIP-1a (Supplemental Fig.  1C), and MCP-1 (Supplemental Fig.  1D) were significantly increased in the sera of CRC patients compared to healthy controls. The distribution frequencies of the 8,061 genes in terms of their Spearman correlations with eotaxin-1 levels are shown in Figure 3(a). Notably, the expression levels of all 8,061 genes correlated with serum eotaxin-1 concentrations. A total of 1,063 of these genes were involved in cell adhesion, inflammation, and the immune response (e.g., MHC, CD1d, TLR4, IL-15, Fc gamma, and Hck; Table 1). A total of 974 genes, the expression levels of which were negatively correlated with eotaxin-1 concentrations, were involved in proteolysis, development, and reproduction (Table 2). These biological processes are characteristics of monocytes and macrophages rather than T cells. The distribution frequencies of the 8,061 genes in terms of their Spearman correlations with serum IL-8 concentrations are shown in Figure 3(b). Almost all genes were so correlated. A total of 250 genes expressed in peripheral CD4+ cells were positively correlated with the serum IL-8 concentration, the genes played roles in cell adhesion, inflammation, the immune response, cytoskeletal processes, and development (Table 3). The expression levels of 586 genes were negatively correlated with serum IL-8 concentration; these genes were involved in cell proliferation, development, and reproduction (Table 4). These biological processes were also characteristic of monocytes and macrophages, rather than T cells. Overall, the serum levels of eotaxin-1 and IL-8 in CRC patients substantially correlated with the expression levels of certain genes in peripheral CD4+ cells compared to healthy controls.

## 4. Discussion

Based on our previous findings that the immune pathophysiology of digestive system cancers is reflected in peripheral blood, we investigated the inflammatory conditions of CRC patients by assessing cytokine/chemokine and performing gene expression analyses of peripheral blood using bead-based multiplex immunoassay and DNA microarray, respectively. Gene expression in peripheral CD4+ and whole blood cells differed between CRC patients and healthy controls [5]. The serum levels of eotaxin-1 and IL-8 were significantly elevated in CRC patients, and the levels significantly correlated with changes in the gene expression levels in CD4+ cells.

Cytokines/chemokines (humoral immunomodulators) regulate cancer-associated immune responses [15]. Eotaxin-1 (CCL11) controls both the eosinophil-mediated immune response [16] and other immune responses of Th2 cells [17]. We found that eotaxin-1 levels were elevated in the sera of CRC patients. The expression levels of certain other genes that significantly differed between peripheral CD4+ cells of CRC patients and those of healthy controls also correlated with serum eotaxin-1 concentrations. The upregulated genes, including those encoding MHC, CD1d, TLR4, IL-15, Fc gamma, and Hck [18], are normally expressed in monocytes/macrophages. In humans, both T cells and monocytes express CD4; however, the cell functions differ [19].

Serum IL-8 (CXCL8) levels were significantly elevated in CRC patients and those with other cancers [20–22]. IL-8 fosters CRC tumor growth, invasion, and metastasis [23, 24], promoting* in vitro *cell proliferation of human colon carcinoma cells via metalloproteinase-mediated cleavage [25]. Additionally, tumor-derived IL-8 induces the formation of immunosuppressive neutrophils and myeloid-derived suppressor cells in tumor microenvironments [26, 27]. Thus, elevated serum IL-8 levels in CRC patients may play an important role in cancer progression; indeed, attainment of an advanced clinical stage was associated with an increase in serum IL-8 concentration. Serum IL-8 levels correlated with changes in the expression levels of CD4+ cell genes compared to healthy controls; these changes also suggested that phagocytosis was in play.

Because immune-mediating cells are miscellaneous, including myeloid-derived cells such as neutrophils, monocytes, and lymphocytes, the interaction of these immune-mediating cells in CRC should be studied to further understand the immune pathophysiological features of CRC. The most frequent subpopulation of whole blood cells is neutrophils. We observed that the gene expression profile of whole blood cells and CD4+ cells was discernible between CRC patients and healthy volunteers; thus, the interaction between these two populations should particularly be investigated.

Collectively, this study showed that transcriptional alteration of peripheral blood, especially CD4+ cells, and elevation of humoral mediators were possibly reflection of immune pathophysiology of CRC, which are compatible to the recent other reports showing gene expression profile alteration [28–30] as well as alteration of concentration of humoral immune mediators [31] in peripheral blood. Humoral immune mediators and cellular immunity are interactive [32, 33]. As the immune system and its reaction are extremely complex, especially in cancers [34], each humoral mediator and cellular fraction should be further investigated to understand immune pathophysiology in detail. Despite these possible immune pathophysiological features being reflected by serum chemokines and peripheral CD4+ cells, further analysis in a larger cohort than that used in the current study should be performed to explore interactive features between chemokines, eotaxin-1 and IL-8, and CD4+ cells in peripheral blood of CRC patients.

In conclusion, we showed that CRC featured systemic inflammation, changes in the serum concentrations of eotaxin-1 and IL-8, and correlated changes in gene expression in peripheral blood CD4+ cells. Further studies exploring the roles played by chemokines and peripheral CD4+ cells in CRC patients are required. In addition, it should be explored how eotaxin-1 and IL-8 elevation is correlated with clinical outcome of CRC in terms of overall survival, therapeutic response after curative treatment with endoscopy or surgery, and relapse rate after complete cure.

## Figures and Tables

**Table 1:** Biological process networks for 1063 genes whose expression in peripheral CD4+ cells was positively correlated with serum eotaxin concentration.

Networks	Total	*P *value	False discovery rate	In data	Network objects from active data
Immune response_Phagocytosis	222	2.27E-08	1.81E-06	33	ITGB2, Syk, IL-15, RhoA, Myosin I, C/EBP, Dectin-1, Fc gamma RII beta, ILT4, MSN (moesin), ERM proteins, SHPS-1, Fc epsilon RI gamma, MSR1, MANR, Lyn, PLC-gamma 2, IL-15RA, Btk, ILT2, Hck, TLR4, MARCO, MARCKS, MLCK, PLC-gamma, gp91-phox, p40-phox, PAK1, p67-phox, FGR, Fc alpha receptor, Fc gamma RII alpha

Cell adhesion_Platelet aggregation	158	2.34E-08	1.81E-06	27	ITGB2, Syk, GAB2, PLA2, RhoA, Thrombospondin 1, G-protein beta/gamma, COX-1 (PTGS1), Fc epsilon RI gamma, c-Src, ENP1, THAS, cPLA2, PTAFR, Lyn, PLC-gamma 2, PKA-reg (cAMP-dependent), GP-IX, P2Y12, Gab, G-protein alpha-i family, G-protein alpha-i2, P2X1, MLCK, PLC-gamma, CD36, VAV-2

Cell adhesion_Amyloid proteins	195	6.02E-06	3.09E-04	26	RhoA, FZD1, Nicastrin, NOTCH2, APLP2 active fragment, G-protein beta/gamma, Jagged1, Nidogen, p120-catenin, Tcf(Lef), c-Src, Presenilin 2, FZD2, FZD5, Notch, Presenilin 1, Alpha-catenin, APLP2 precursor, Cathepsin D, MALS, Frizzled, ADAM9, PKC, PAK1, Plakoglobin, Presenilin

Immune response_Antigen presentation	197	6.23E-05	2.40E-03	24	CIITA, ICAM1, MHC class II beta chain, CD1b, HLADPA1, CD1d, HLA-DQA1, JAK2, HA2Z, Fc epsilon RI gamma, IP-30, HLA-DM, HLA-DRB1, CD1a, Cathepsin S, LFA-3, MHC class II, HLA-DQB1, HLA-DRA1, HLA-DPB1, HLA-DRB4, CD86, CD74, RING6

Proliferation_Positive regulation cell proliferation	221	1.48E-04	4.56E-03	25	p21, HGF, RhoG, Beta-arrestin1, GAB2, Fra-1, RhoA, Galpha(i)-specific peptide GPCRs, JAK2, MTG16 (CBFA2T3), c-Src, VEGF-A, RasGRP4, PKA-reg (cAMP-dependent), G-protein alpha-15, TCIRG1 (Atp6i), CCR1, G-protein alpha-i family, M-CSF receptor, G-protein alpha-i2, CSDA, MLCK, c-Fes, FLT3, PAK1

Chemotaxis	137	2.00E-04	5.14E-03	18	ITGB2, Syk, C5aR, Fra-1, GRO-2, Prokineticin 2, PD-ECGF (TdRPase), IL-1 beta, Galpha(i)-specific peptide GPCRs, PLAUR (uPAR), MIG, Integrin, VEGF-A, PTAFR, CCR1, G-protein alpha-i family, Galpha(q)-specific peptide GPCRs, PLD1

Inflammation_IFN-gamma signaling	109	4.08E-04	8.97E-03	15	CIITA, p21, ITGB2, IL-15, IL-18, ICAM1, PKC-delta, K12, JAK2, MIG, c-Src, PLC-gamma 2, TLR4, PLC-gamma, Fc alpha receptor

Apoptosis_Anti-apoptosis mediated by external signals via NF-kB	111	4.97E-04	9.57E-03	15	IL-15, MyD88, G-protein beta/gamma, TNF-R2, VEGF-A, CD30(TNFRSF8), CSF2RA, PKA-reg (cAMP-dependent), IL-15RA, G-protein alpha-i family, TLR4, Bcl-3, TL1A(TNFSF15), APRIL(TNFSF13), BAFF(TNFSF13B)

Inflammation_Neutrophil activation	215	6.08E-04	1.02E-02	23	ITGB2, C5aR, PLA2, ICAM1, GRO-2, RhoA, PKC-delta, G-protein beta/gamma, TNF-R2, Galpha(i)-specific peptide GPCRs, Syntaxin 7, cPLA2, Btk, G-protein alpha-15, G-protein alpha-i family, G-protein alpha-i2, PA24A, gp91-phox, ALOX5, p40-phox, PAK1, p67-phox, PLD1

Inflammation_IL-4 signaling	115	7.27E-04	1.02E-02	15	HLADPA1, HLA-DQA1, JAK2, MHC class II, Bax, HLA-DQB1, HLA-DRA1, HLA-DPB1, HLA-DRB4, CD86, CD74, c-Fes, CD13, IL13RA1, Fc gamma RII alpha

**Table 2:** Biological process networks for 974 genes whose expression was negatively correlated with CD4+ peripheral blood cells and Eotaxin.

Networks	Total	*P *value	False discovery rate	In data	Network objects from active data
Proteolysis_ECM remodeling	85	5.38E-05	7.21E-03	10	Collagen XIV, Tenascin-C, NEPH2, MMP-16, Protein C inhibitor, Serpin B12, COL18A1, Kallikrein 2, Trypsin II, Aggrecanase-1

Neurophysiological process_Transmission of nerve impulse	212	3.13E-04	2.10E-02	15	L-type Ca(II) channel, alpha 1C subunit, GABA-A receptor gamma-2 subunit, KCC2, mGluR3, Galpha(i)-specific peptide GPCRs, mGluR1, Galpha(q)-specific metabotropic glutamate GPCRs, Ionotropic glutamate receptor, Galpha(i)-specific metabotropic glutamate GPCRs, GluR6, Galpha(i)-specific amine GPCRs, CHT1, RIN, G-protein alpha-s, Kainate receptor

Reproduction_Gonadotropin regulation	199	1.64E-03	5.29E-02	13	L-type Ca(II) channel, alpha 1C subunit, GABA-A receptor gamma-2 subunit, mGluR3, mGluR1, Galpha(q)-specific metabotropic glutamate GPCRs, Ionotropic glutamate receptor, Galpha(i)-specific metabotropic glutamate GPCRs, Secretogranin 1, Protein kinase G1, Adenylate cyclase, G-protein alpha-s, Protein kinase G, Kainate receptor

Development_Blood vessel morphogenesis	228	1.97E-03	5.29E-02	14	PDE, Galpha(i)-specific peptide GPCRs, PDE7A, Endomucin, Galpha(q)-specific amine GPCRs, Galpha(i)-specific amine GPCRs, Protein kinase G1, Galpha(q)-specific peptide GPCRs, COL18A1, Tissue kallikreins, Neuropilin-1, G-protein alpha-s, Protein kinase G, Transferrin

Reproduction_Spermatogenesis, motility and copulation	228	1.97E-03	5.29E-02	14	PDGF receptor, MFGE8, IGF-1 receptor, Ropporin, MSK1, S5AR2, BBS2, Tissue kallikreins, BMP2, Kallikrein 2, SOX5, CREM (activators), ZFP37, PDGF-R-alpha

Proteolysis_Connective tissue degradation	119	3.23E-03	6.80E-02	9	Trypsin, Tenascin-C, MMP-16, Protein C inhibitor, Serpin B12, Tissue kallikreins, Kallikrein 2, Trypsin II, Aggrecanase-1

Development_Neurogenesis in general	192	3.55E-03	6.80E-02	12	WNT4, RET, CHRM, Neuromodulin, WNT7A, WNT, Galpha(q)-specific amine GPCRs, Galpha(i)-specific amine GPCRs, HDAC7, ACM3, SOX8, SOX14

Development_Cartilage development	66	6.45E-03	1.08E-01	6	TR-alpha, Noggin, COL1A2, BMP2, SOX5, Aggrecanase-1

Reproduction_Male sex differentiation	243	8.98E-03	1.30E-01	13	AP-2A, PDGF receptor, Olfactory receptor, RET, IGF-1 receptor, MSK1, S5AR2, HSF2, BMP2, SOX5, CREM (activators), ZFP37, PDGF-R-alpha

Reproduction_GnRH signaling pathway	166	9.70E-03	1.30E-01	10	GABA-A receptor gamma-2 subunit, mGluR3, mGluR1, Galpha(q)-specific metabotropic glutamate GPCRs, Ionotropic glutamate receptor, Galpha(i)-specific metabotropic glutamate GPCRs, Protein kinase G1, G-protein alpha-s, Protein kinase G, Kainate receptor

**Table 3:** Biological process networks for 250 genes whose expression in peripheral CD4+ cells was positively correlated with serum IL-8 concentration.

Networks	Total	*P *value	False discovery rate	In data	Network objects from active data
Cytoskeleton_Actin filaments	176	2.12E-05	2.31E-03	10	Actin muscle, Talin, Tropomyosin, RhoA, Myosin I, CAPZA, MELC, TARA, Actin, CAPZA1

Cytoskeleton_Regulation of cytoskeleton rearrangement	183	8.79E-04	3.67E-02	8	Actin muscle, Talin, RhoA, CAPZA, MELC, TARA, Actin, CAPZA1

Development_Skeletal muscle development	144	1.01E-03	3.67E-02	7	ACTA2, Smooth muscle myosin, Actin muscle, Tropomyosin, RhoA, MELC, Actin

Muscle contraction	173	2.90E-03	6.54E-02	7	ACTA2, Syntrophin B, Smooth muscle myosin, Actin muscle, Tropomyosin, MELC, Actin

Immune response_Phagocytosis	222	3.00E-03	6.54E-02	8	ILT2, Talin, CD63, RhoA, Myosin I, MSR1, MELC, Actin

Cell adhesion_Integrin priming	110	7.22E-03	1.17E-01	5	ACTA2, ITGA2B, Talin, Integrin, Actin

Cell adhesion_Platelet aggregation	158	7.84E-03	1.17E-01	6	COX-1 (PTGS1), Talin, RhoA, ENP1, MELC, GP-IB beta

Cell adhesion_Integrin-mediated cell-matrix adhesion	214	9.17E-03	1.17E-01	7	ITGA2B, ITGB5, Talin, Integrin, RhoA, MELC, Actin

Inflammation_Amphoterin signaling	118	9.64E-03	1.17E-01	5	ITGAM, MyD88, RhoA, MELC, Actin

Proteolysis_Proteolysis in cell cycle and apoptosis	125	1.22E-02	1.33E-01	5	Presenilin 2, Cathepsin C, FBX6, Pseudo-ICE, Presenilin

**Table 4:** Biological process networks for 586 genes whose expression in peripheral CD4+ cells was negatively correlated with serum IL-8 concentration.

Networks	Total	*P *value	False discovery rate	In data	Network objects from active data
Muscle contraction	173	2.69E-03	1.78E-01	7	K(+) channel, subfamily J, Dystrophin, PKC-alpha, MyHC, PKC, Titin, cPKC (conventional)

Development_Blood vessel morphogenesis	228	3.26E-03	1.78E-01	8	PDE3B, PKC-alpha, COL18A1, PDE, PDE9A, TERT, PDE7A, Endomucin

Cardiac development_Wnt_beta-catenin, Notch, VEGF, IP3 and integrin signaling	150	5.75E-03	2.09E-01	6	PKC-alpha, Polycystin, CHIBBY, MyHC, Titin, LRP6

Cardiac development_FGF_ErbB signaling	124	1.12E-02	3.05E-01	5	PKC-alpha, Polycystin, MyHC, Titin, gp130

Development_Skeletal muscle development	144	2.02E-02	4.41E-01	5	Dystrophin, HDAC7, Histone deacetylase class II, MyHC, Titin

Proliferation_Positive regulation cell proliferation	221	3.32E-02	5.15E-01	6	PUR-alpha, RASGRF1, COL18A1, IGF-1 receptor, IL-11 receptor, gp130

Development_Cartilage development	66	3.51E-02	5.15E-01	3	TR-alpha, Noggin, Aggrecanase-1

Reproduction_Spermatogenesis, motility and copulation	228	3.78E-02	5.15E-01	6	MSK1, MFGE8, Oct-3/4, IGF-1 receptor, PKC, ZFP37

Reproduction_Male sex differentiation	243	4.90E-02	5.41E-01	6	MSK1, Oct-3/4, IGF-1 receptor, PKC, ZFP37, PMEPA1

Transcription_Chromatin modification	127	4.97E-02	5.41E-01	4	SATB1, MSK1, HDAC7, Histone deacetylase class II

## Data Availability

The datasets used and/or analyzed during the current study are available from the corresponding author on reasonable request.

## Supplementary Materials

Supplementary 1 Supplemental Table 1. Characteristics of serum chemokine study subjects.

Supplementary 2 Supplemental Table 2. Characteristics of study subjects.

Supplementary 3 Supplemental Fig. 1. Serum concentrations of cytokines and chemokines.

Supplementary 4 Supplemental Fig. 2. Serum concentrations of cytokines and chemokines.
