# Supplementary material for: Immune Condition of Colorectal Cancer Patients Featured by Serum Chemokines and Gene Expressions of CD4+ Cells in Blood
Source: Can J Gastroenterol Hepatol. 2018 Jun 11;2018:7436205. doi: 10.1155/2018/7436205 (PMC6016223; doi:10.1155/2018/7436205)
Supplement: Supplementary 1 — Supplemental Table 1. Characteristics of serum chemokine study subjects. [file 7436205.f1.pptx]

## Slide 1
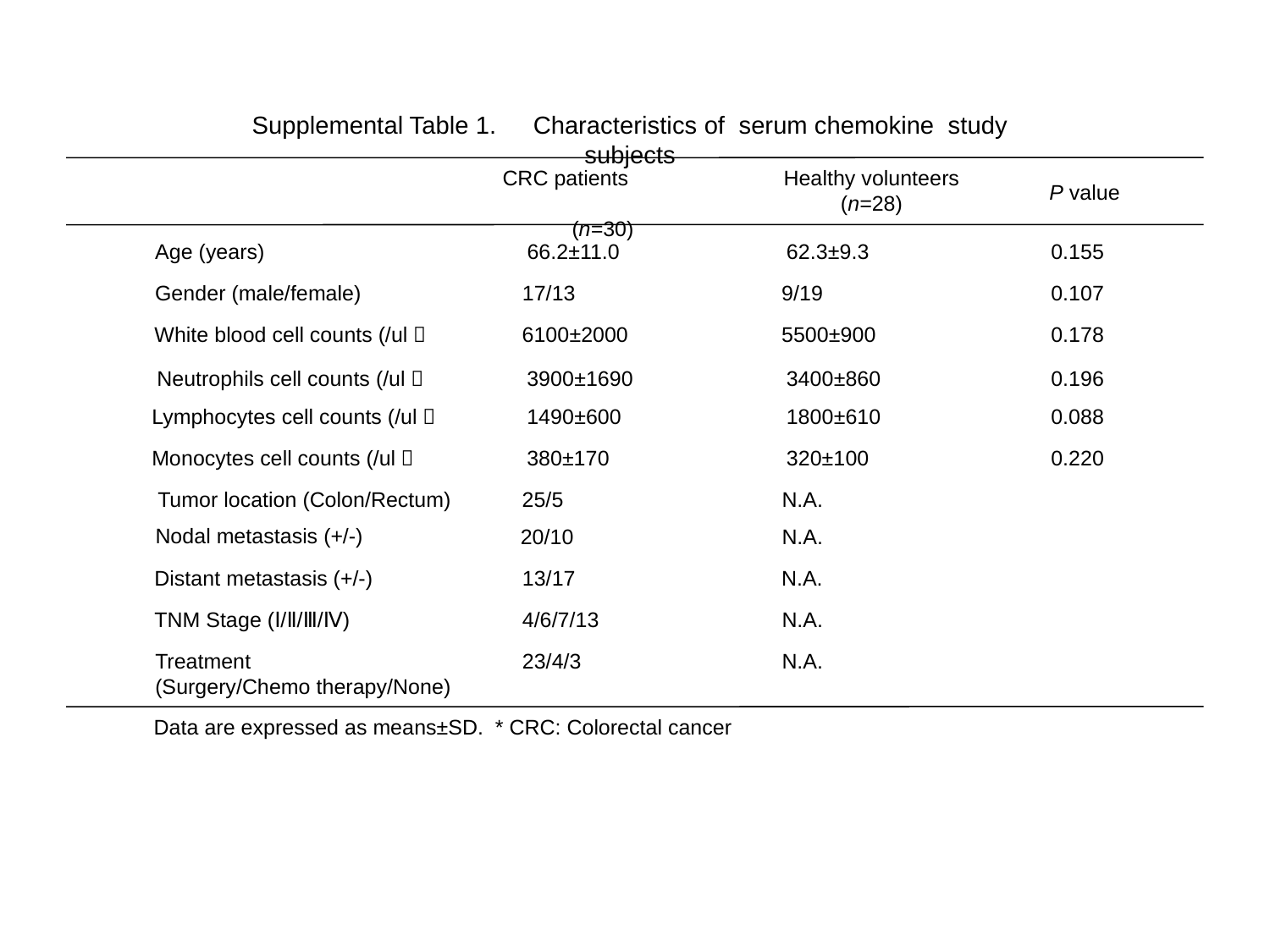

Supplemental Table 1.　Characteristics of serum chemokine study subjects
CRC patients
(n=30)
Healthy volunteers
(n=28)
P value
Age (years)
66.2±11.0
62.3±9.3
0.155
Gender (male/female)
17/13
9/19
0.107
White blood cell counts (/ul）
6100±2000
5500±900
0.178
Neutrophils cell counts (/ul）
3900±1690
3400±860
0.196
Lymphocytes cell counts (/ul）
1490±600
1800±610
0.088
Monocytes cell counts (/ul）
380±170
320±100
0.220
Tumor location (Colon/Rectum)
25/5
N.A.
Nodal metastasis (+/-)
20/10
N.A.
Distant metastasis (+/-)
13/17
N.A.
TNM Stage (Ⅰ/Ⅱ/Ⅲ/Ⅳ)
4/6/7/13
N.A.
Treatment
(Surgery/Chemo therapy/None)
23/4/3
N.A.
Data are expressed as means±SD. * CRC: Colorectal cancer
