# Supplementary material for: Immune Condition of Colorectal Cancer Patients Featured by Serum Chemokines and Gene Expressions of CD4+ Cells in Blood
Source: Can J Gastroenterol Hepatol. 2018 Jun 11;2018:7436205. doi: 10.1155/2018/7436205 (PMC6016223; doi:10.1155/2018/7436205)
Supplement: Supplementary 2 — Supplemental Table 2. Characteristics of study subjects. [file 7436205.f2.pptx]

## Slide 1
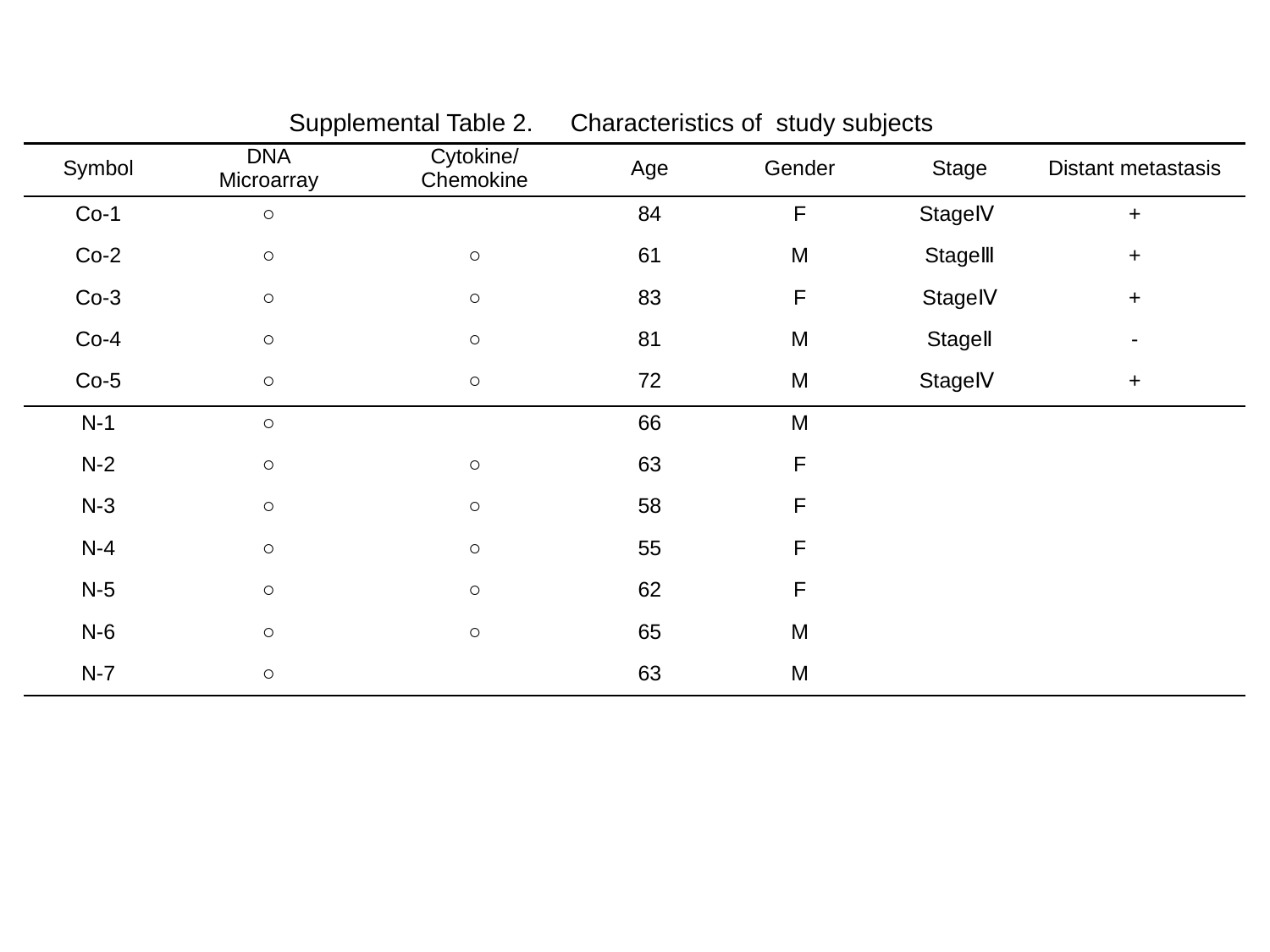

Supplemental Table 2.　Characteristics of study subjects
| Symbol | DNA Microarray | Cytokine/ Chemokine | Age | Gender | Stage | Distant metastasis |
| --- | --- | --- | --- | --- | --- | --- |
| Co-1 | ○ | | 84 | F | StageⅣ | + |
| Co-2 | ○ | ○ | 61 | M | StageⅢ | + |
| Co-3 | ○ | ○ | 83 | F | StageⅣ | + |
| Co-4 | ○ | ○ | 81 | M | StageⅡ | - |
| Co-5 | ○ | ○ | 72 | M | StageⅣ | + |
| N-1 | ○ | | 66 | M | | |
| N-2 | ○ | ○ | 63 | F | | |
| N-3 | ○ | ○ | 58 | F | | |
| N-4 | ○ | ○ | 55 | F | | |
| N-5 | ○ | ○ | 62 | F | | |
| N-6 | ○ | ○ | 65 | M | | |
| N-7 | ○ | | 63 | M | | |
| | | | | | | |
