# Supplementary material for: Immune Condition of Colorectal Cancer Patients Featured by Serum Chemokines and Gene Expressions of CD4+ Cells in Blood
Source: Can J Gastroenterol Hepatol. 2018 Jun 11;2018:7436205. doi: 10.1155/2018/7436205 (PMC6016223; doi:10.1155/2018/7436205)
Supplement: Supplementary 3 — Supplemental Fig. 1. Serum concentrations of cytokines and chemokines. [file 7436205.f3.pptx]

## Slide 1
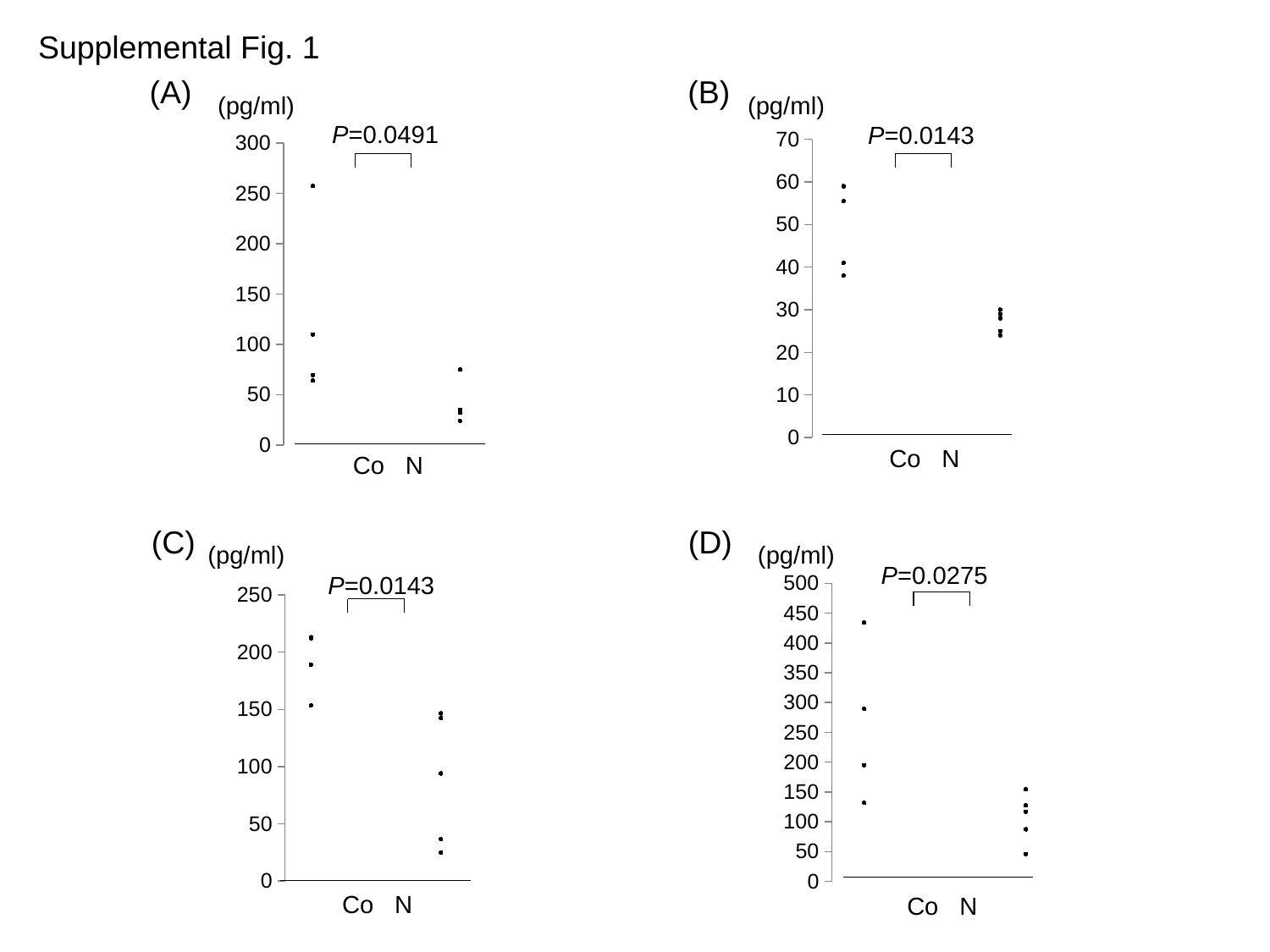

Supplemental Fig. 1
(A)
(B)
(pg/ml)
(pg/ml)
P=0.0491
P=0.0143
### Chart
| Category | |
|---|---|
### Chart
| Category | |
|---|---|Co
N
Co
N
(C)
(D)
(pg/ml)
(pg/ml)
P=0.0275
### Chart
| Category |
|---|P=0.0143
### Chart
| Category | |
|---|---|
### Chart
| Category | |
|---|---|
### Chart
| Category |
|---|Co
N
Co
N
