# Supplementary material for: Immune Condition of Colorectal Cancer Patients Featured by Serum Chemokines and Gene Expressions of CD4+ Cells in Blood
Source: Can J Gastroenterol Hepatol. 2018 Jun 11;2018:7436205. doi: 10.1155/2018/7436205 (PMC6016223; doi:10.1155/2018/7436205)
Supplement: Supplementary 4 — Supplemental Fig. 2. Serum concentrations of cytokines and chemokines. [file 7436205.f4.pptx]

## Slide 1
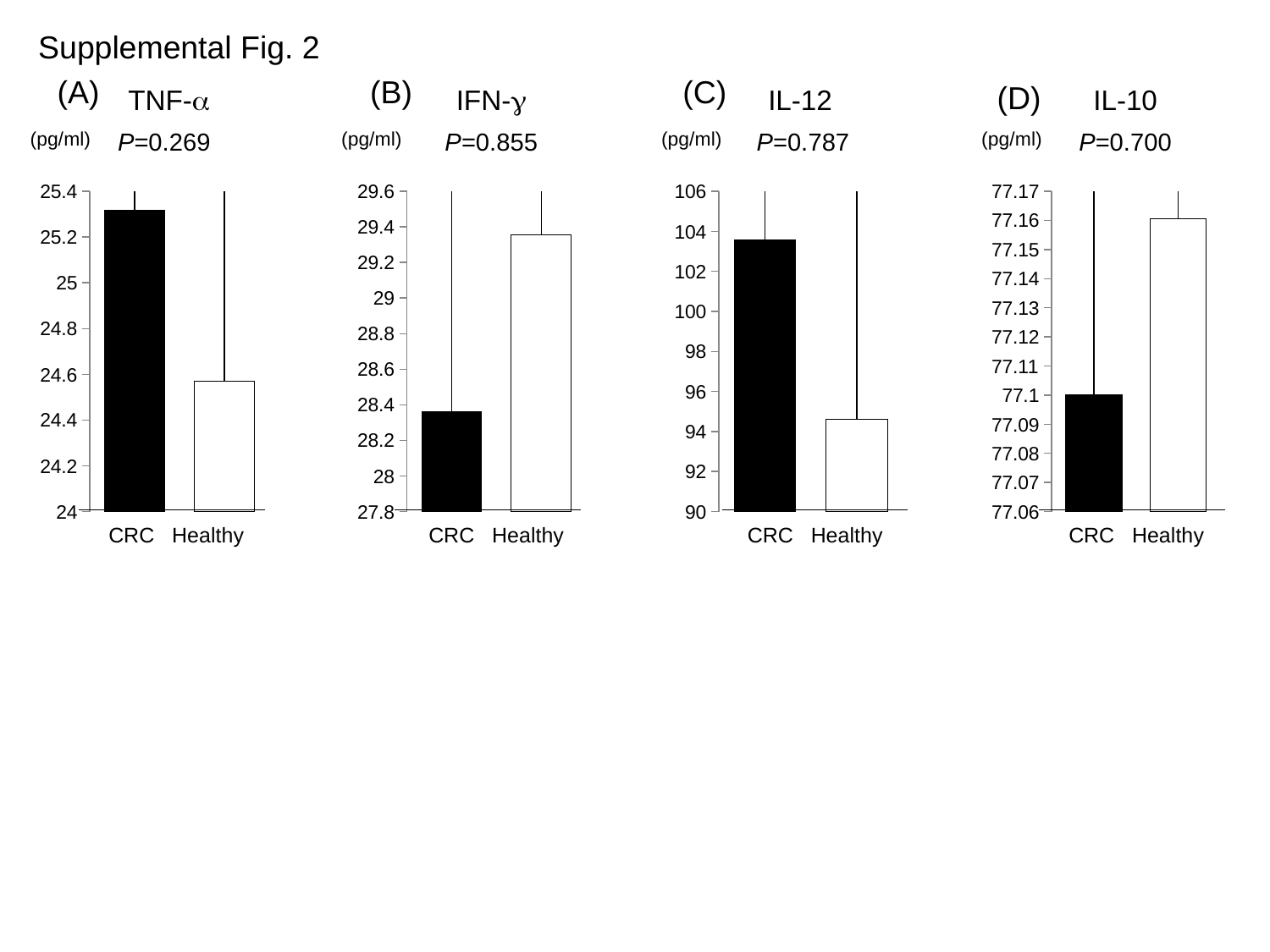

(b)
Supplemental Fig. 2
(A)
(B)
(C)
(D)
TNF-a
IFN-g
IL-12
IL-10
(pg/ml)
P=0.269
(pg/ml)
P=0.855
(pg/ml)
P=0.787
(pg/ml)
P=0.700
### Chart
| Category | 平　均 |
|---|---|
| colon ca | 25.316666666666666 |
| Healthy | 24.571428571428573 |
### Chart
| Category | 平　均 |
|---|---|
| colon ca | 28.359375 |
| Healthy | 29.357142857142858 |
### Chart
| Category | 平　均 |
|---|---|
| colon ca | 103.58333333333333 |
| Healthy | 94.58928571428571 |
### Chart
| Category | 平　均 |
|---|---|
| colon ca | 77.1 |
| Healthy | 77.16071428571429 |CRC Healthy
CRC Healthy
CRC Healthy
CRC Healthy
